# CBD enhances the cognitive score of adolescent rats prenatally exposed to THC and fine-tunes relevant effectors of hippocampal plasticity

**DOI:** 10.3389/fphar.2023.1237485

**Published:** 2023-07-31

**Authors:** Valentina Castelli, Gianluca Lavanco, Cesare D’Amico, Salvatore Feo, Giuseppe Tringali, Martin Kuchar, Carla Cannizzaro, Anna Brancato

**Affiliations:** ^1^ Department of Biomedicine, Neuroscience and Advanced Diagnostics, University of Palermo, Palermo, Italy; ^2^ Department of Health Promotion, Mother and Child Care, Internal Medicine and Medical Specialties of Excellence “G. D’Alessandro”, University of Palermo, Palermo, Italy; ^3^ Department of Biological, Chemical and Pharmaceutical Sciences and Technologies and ATEN Center, University of Palermo, Palermo, Italy; ^4^ Pharmacology Section, Department of Healthcare Surveillance and Bioethics, Università Cattolica del Sacro Cuore, Rome, Italy; ^5^ Fondazione Policlinico Universitario A. Gemelli IRCSS, Rome, Italy; ^6^ Forensic Laboratory of Biologically Active Compounds, Department of Chemistry of Natural Compounds, University of Chemistry and Technology, Prague, Czechia; ^7^ Psychedelics Research Centre, National Institute of Mental Health, Prague, Czechia

**Keywords:** cannabidiol, prenatal THC exposure, adolescent offspring, spatial memory, hippocampal excitatory synapse, CB1R

## Abstract

**Introduction:** An altered neurodevelopmental trajectory associated with prenatal exposure to ∆-9-tetrahydrocannabinol (THC) leads to aberrant cognitive processing through a perturbation in the effectors of hippocampal plasticity in the juvenile offspring. As adolescence presents a unique window of opportunity for “brain reprogramming”, we aimed at assessing the role of the non-psychoactive phytocannabinoid cannabidiol (CBD) as a rescue strategy to temper prenatal THC-induced harm.

**Methods:** To this aim, Wistar rats prenatally exposed to THC (2 mg/kg s.c.) or vehicle (gestational days 5–20) were tested for specific indexes of spatial and configural memory in the reinforcement-motivated Can test and in the aversion-driven Barnes maze test during adolescence. Markers of hippocampal excitatory plasticity and endocannabinoid signaling—NMDAR subunits NR1 and 2A-, mGluR5-, and their respective scaffold proteins PSD95- and Homer 1-; CB1R- and the neuromodulatory protein HINT1 mRNA levels were evaluated. CBD (40 mg/kg i.p.) was administered to the adolescent offspring before the cognitive tasks.

**Results:** The present results show that prenatal THC impairs hippocampal memory functions and the underlying synaptic plasticity; CBD is able to mitigate cognitive impairment in both reinforcement- and aversion-related tasks and the neuroadaptation of hippocampal excitatory synapses and CB1R-related signaling.

**Discussion:** While this research shows CBD potential in dampening prenatal THC-induced consequences, we point out the urgency to curb cannabis use during pregnancy in order to avoid detrimental bio-behavioral outcomes in the offspring.

## 1 Introduction

The rapidly evolving legal and social framework around the use of cannabis results in the rise of cannabis as the most commonly consumed illicit drug in pregnancy for symptom management, such as first-trimester nausea (Nguyen and Harley, 2022; Taneja et al., 2022). However, the perception of benefits should be weighed against the harm risk of gestational cannabis exposure. ∆-9-Tetrahydrocannabinol (THC), the primary psychoactive constituent of cannabis, crosses the placenta and interfaces with the endocannabinoid (eCB) system (ECS) in the fetal brain where cannabinoid receptors type 1 (CB1Rs) are already expressed in high levels in cortical regions and the hippocampus since the first trimester (Wang et al., 2003; Habayeb et al., 2008; Chan et al., 2013; Paul et al., 2021). Indeed, the ECS, which mediates the actions of THC, plays a critical regulatory role throughout all developmental stages, from neurogenesis and neuronal migration to the regulation of signaling pathways and synaptic transmission (Basavarajappa et al., 2009; Meyer and Gee, 2018). Synaptic plasticity, in particular, is critically dependent on the strength, integrity and structural organization of the synaptic connections to maintain and support the related functions. In particular, hippocampal synaptic plasticity is postulated to be an important cellular substrate for the encoding and storage of associative, long-term spatial memories (Bannerman et al., 2014; Goto, 2022). Given these premises, it is more and more evident that in-utero exposure to cannabis can represent a threat to the regular development of the morphological and functional hippocampal architecture (Thomason et al., 2021). Indeed, observations of deficits in learning, memory, attention, and aggressive behavior have been assessed in children and adolescents exposed to maternal cannabis use (Fried and Smith, 2001; Huizink and Mulder, 2006; Wu et al., 2011; Huizink, 2014; Brancato and Cannizzaro, 2018). Recently, the human behavioral outcomes have been paralleled by preclinical evidence of cognitive deficits paired with a complex rearrangement in the hippocampal excitatory synapse and eCBs signalosome of the adolescent offspring (Wu et al., 2011; Wei and Piomelli, 2015; Castelli et al., 2023; Peng et al., 2023). Indeed, the role of the ECS is to modulate transient- and long-lasting changes in hippocampal synaptic strength (Winter et al., 2021) by controlling both excitatory and inhibitory output, thus supporting learning and memory. For instance, when it is required in the glutamatergic synapse, by the engagement of mGluR5 and the long-isoform Homer scaffold protein, eCBs are released and bind to CB1Rs, thus long-term depression (LTD) occurs (Xu and Chen, 2015; Busquets-Garcia et al., 2018). Additionally, a concerted activity of the histidine triad nucleotide-binding protein 1 (HINT1) physically couples CB1R and NMDAR NR1 subunit (Sánchez-Blázquez et al., 2014; Vicente-Sánchez et al., 2013) to prevent NMDAR over-activation. On the other hand, eCBs signaling in inhibitory synapses would occur through CB1Rs in GABAergic neurons, which would decrease GABA release, disinhibiting postsynaptic neurons in the hippocampus (Selvam et al., 2018). It has been shown by several groups that an imbalance in the inhibitory/excitatory network plays a crucial role in the detrimental cognitive effects exerted by prenatal cannabis exposure in adult mice offspring and adolescent rats, likely as a result of the early interference of THC on the ECS-mediated shaping of hippocampal neural network activity (Vargish et al., 2017; de Salas-Quiroga et al., 2020; Castelli et al., 2023).

Similar to early life, early adolescence is a distinct period of neural maturation and plasticity, wherein a significant amount of refinement occurs, specifically in areas relevant to cognitive function, reinforce, and emotionality (Spear, 2013; Patel et al., 2021). Therefore, it may represent a window of opportunity for programming interventions aimed at tempering the harm of prenatal exposure to THC in the brain. In this context, over the last decade, interest in cannabidiol (CBD) has emerged (Broyd et al., 2016; Osborne et al., 2017; Crippa et al., 2018). Indeed, besides its therapeutic activity as an adjunctive treatment in schizophrenia, social anxiety, pain, and depression (Masataka, 2019; García-Gutiérrez et al., 2020; Oberbarnscheidt and Miller, 2020; Hameed et al., 2023), CBD is safely employed in the pediatric population for treating refractory epilepsy (Raucci et al., 2020). Moreover, emerging evidence suggests that CBD ameliorates learning impairment (Solowij et al., 2018), elicits memory-rescuing effects in various neurodegenerative diseases (Fagherazzi et al., 2012; Bhunia et al., 2022), and improves spatial- and vocal learning following brain damage (Schiavon et al., 2014; Alalawi et al., 2019; Aychman et al., 2023). However, the real spectrum of its clinical effectiveness and pharmacodynamics remains a field of investigation (Sholler et al., 2020).

Therefore, our study aimed at assessing the effects of CBD administration on the cognitive score of prenatally THC-exposed adolescent rat offspring, both in a reinforcement- and an aversion-related context. Moreover, we tested CBD as a rectifying strategy for the prenatal THC-induced alterations in the effectors of hippocampal synaptic plasticity that underlie memory processing. They include: ionotropic glutamate N-methyl-d-aspartate receptors (NMDARs) subunits, such as NR1—necessary for the stable channel folding, assembly, and receptor sensitivity (Flores-Soto et al., 2012), and NR2A—critical for determining the polarity of excitatory synaptic plasticity (Liu et al., 2004); scaffolding protein post-synaptic density-95 (PSD95)—a determinant of the functional diversity and clustering of NMDARs at synaptic sites (Keith and El-Husseini, 2008); group I metabotropic glutamate receptors 5 (mGluRs5)- and scaffolding protein Homer 1, which cooperate to promote the occurring of eCBs-mediated LTD of the synaptic strength (Shiraishi-Yamaguchi et al., 2007; Batista et al., 2016; Castelli et al., 2017); CB1Rs and histidine triad nucleotide-binding protein 1 (HINT1), able to strictly cooperate with CB1Rs to buffer NR1 excessive expression and the related glutamatergic hyperactivation (Sánchez-Blázquez et al., 2014; Rodríguez-Muñoz et al., 2015). The evidence of this research, by adding further developmental brain molecular data, contributes to bridging the gap between prenatal THC exposure and the occurrance of detrimental behavioral outcomes. Moreover, the rescue potential of CBD may help to highlight the role of discrete signaling pathways in modulating the detrimental outcomes following prenatal THC exposure and pinpoint adolescence as a unique sensitive time for brain reprogramming.

## 2 Material and methods

### 2.1 Animals and treatment

Eighteen adult female pregnant Wistar rats at gestational day (GD) 4 (200–220 g; Envigo, Udine, Italy) were singly housed in standard ventilated cages (40 cm × 60 cm × 20 cm) with bedding, maintained at controlled temperature and humidity (22°C ± 2°C and 55% ± 5%, respectively) on a 12-h light/dark cycle, with food and water *ad libitum*. From GD 5 to 20 they were subcutaneously (s.c.) injected with vehicle (Veh) or THC (2 mg/kg). THC dose was chosen based on previous studies (Brancato et al., 2020a; Brancato et al., 2020b; Castelli et al., 2023) and corresponds to mild THC consumption in humans (Frau et al., 2019). After weaning, male rats were housed in pairs, and each experimental group tested in the cognitive tasks included one or two independent male rats per each litter of Veh- or THC-treated dams. The molecular assessment was carried out on one independent male rat per each litter who underwent behavioral assessments, to avoid the litter effect. All experiments were approved by the Italian Ministry of Health (172/2019-PR to Carla Cannizzaro) and conducted in accordance with animal protocols approved by the Committee for the Protection and Use of Animals of the University of Palermo, in adherence with the current Italian legislation on animal experimentation (D.L. 26/2014) and the European directives (2010/63/EU) on care and use of laboratory animals. Every effort was made to minimise the number of animals used and their suffering.

### 2.2 Drugs

THC resin and CBD were obtained from the Forensic Laboratory of Biologically Active Compounds, Department of Chemistry of Natural Compounds, of the University of Chemistry and Technology of Prague, Czech Republic. THC resin (purity (as determined by HPLC) > 97%) (Drazanova et al., 2019) was dissolved in ethanol at 20% concentration, sonicated for 30 min, and then emulsified in 2% Tween 80 and saline (Castelli et al., 2023). CBD (purity (NMR) > 99%) (Hložek et al., 2017) was emulsified in Tween 80 (1%) and saline and, immediately afterward, administered intraperitoneally (i.p.) at the dose of 40 mg/kg. The dose was chosen based on previous evaluations in rats (Chagas et al., 2013; Umpierrez et al., 2022).

### 2.3 Experimental procedures

**FIGURE 1:**
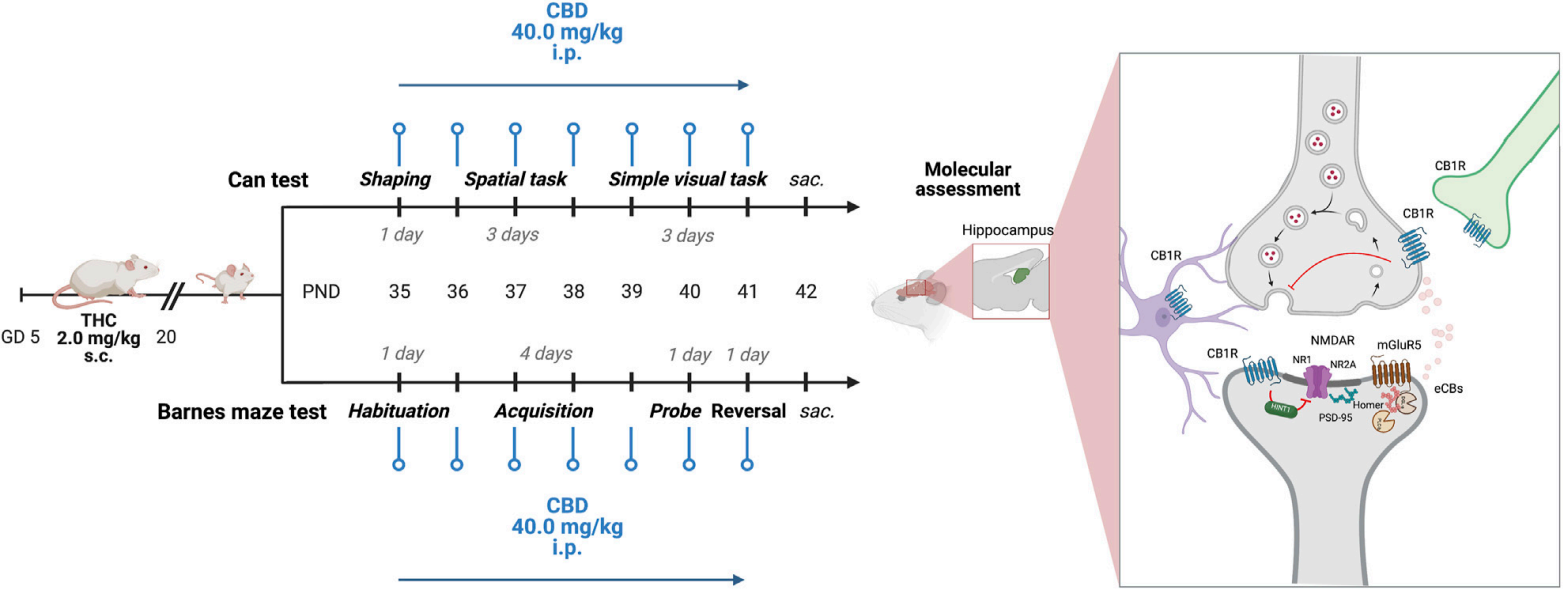
Experimental design. Starting from PND 35, adolescent CTRL and pTHC (2 mg/kg, GD 5–20) rats underwent the spatial- and the simple visual tasks of the Can test or performed the probe- and reversal tasks of the Barnes maze test. CBD (40 mg/kg i.p.) was administered 24 h prior to each experimental session. Animals were sacrificed 24 h after the last experimental task and CBD administration. GD, gestational day; PND, postnatal day; s.c., subcutaneous; i.p., intraperitoneal; THC, Δ-9-tetrahydrocannabinol; CBD, cannabidiol; sac., sacrifice; NR1, ionotropic glutamate N-methyl-d-aspartate receptors (NMDARs) NR1 subunit; NR2A, NMDARs NR2A subunit; PSD95, scaffolding protein post-synaptic density-95; mGluR5, group I metabotropic glutamate receptor 5; CB1R, cannabinoid receptor type 1; HINT1, histidine triad nucleotide-binding protein 1. Created with BioRender.com, https://app.biorender.com (accessed on 15 July 2023).

Adolescent male rat offspring from postnatal day (PND) 35 prenatally exposed to either vehicle (CTRL) or THC (prenatal THC, pTHC) were tested during the light phase of the light/dark cycle. The offspring performed the Can test—a reinforcement-motivated task to assess both spatial- and configural memory (Popoviç et al., 2001)—or was exposed to the Barnes maze test—a mild aversive, dry-land-based behavioral test that allows the assessment of spatial memory and reversal learning in rodents (Gawel et al., 2019). Rats prenatally exposed to THC were administered i.p. with CBD (40 mg/kg) or vehicle 24 h prior to testing the rats for spatial- and configural memory in the Can test or reference memory and reversal learning in the Barnes maze test (Brancato et al., 2021). CTRL rats received vehicle administration on the same days (Figure 1). The behavior of the rats was monitored and quantified by the experimenter and an automatic video-tracking system, AnyMaze (Stoelting Europe, Dublin, Ireland). The objects and apparatus used were thoroughly cleaned at the end of each experimental session.

### 2.4 Can test

The Can test allows the evaluation of spatial and configural cognitive processing in a reinforcement-facilitated context (Popoviç et al., 2001). In the task, rats are motivated to identify a single reinforced can among a set of seven cans. The reinforcement consisted of 0.3 mL tap water placed in the indented bottoms of the can put upside down. A 10-h water deprivation schedule was used as motivation; rats were allowed to drink freely for 1 h at the end of the experimental sessions (Plescia et al., 2014). On the walls of the laboratory room, large colorful geometric figures as visual cues were provided to facilitate the animal’s spatial orientation. The behavioral protocol consisted of three separate phases—shaping period, spatial-, and simple visual tasks—as described below. Cans were painted in white or left in their original colors, according to the task administered, and placed in a square Plexiglas apparatus (100 cm × 100 cm × 43 cm) in a fan-shaped pattern. A “visit” was recorded when the rat stood on its hind paws and brought its nose up to the top edge of the can. The parameters measured were: activity score, i.e., the number of trials during which the rat visited at least one can (up to 10 during each experimental session); correct responses (CR), i.e., the number of trials in which the rat visited the reinforced can first, divided by activity score (up to 1 per each experimental session); reference memory errors (RE), i.e., the first visits to a non-reinforced can on each trial, divided by activity score (up to 6 per each experimental session); working memory errors (WE), i.e., repeated visits to the same non-reinforced can on the same trial divided by the activity score.

In order to outline the reinforcement-related cognitive performance, an integrated z-score was calculated as follows: z = (X–μ)/s, with “s” indicating how many standard deviations the observation (X) is above or below the mean of the control group (μ) (Guilloux et al., 2011; Castelli et al., 2022) based on the spatial- and simple visual tasks of the Can test, using normalization of cognitive parameters, i.e., the numbers of CR, the scores for RE and WE. Individual reinforcement-related cognitive z-scores were then calculated by averaging z-score values. To calculate the recovery impact of CBD, the improvement score related to CR was calculated by subtracting the average of pTHC data (A) from both pTHC- and pTHC-CBD data (B) (delta = B − A) (Lima-Silva et al., 2012). On the other hand, the improvement score referred to RE was computed as delta multiplied by −1.

#### 2.4.1 Experimental design

##### 2.4.1.1 Shaping period

This 2-session phase allowed rats to familiarise themselves with the environment. In the first session, rats were placed in the apparatus with seven white cans, whose bottom was filled with the reinforcement, namely, 0.3 mL tap water. Rats had 10 min to explore the apparatus and take water from the cans. In the second session, two randomly selected cans and the one in the center were reinforced with water. The rats were given up to 10 min to visit and drink water (modified from Popoviç et al., 2001). During a 15-s interval between sessions, rats were placed in a small Plexiglas box.

##### 2.4.1.2 Spatial task

Twenty-four hours after the end of the shaping period, the spatial task took place on 3 consecutive days and along 10 trials per day, in the same environment as the shaping period. The single reinforced can was the one in the center of the apparatus. Rats could spend up to 180 s each trial in order to visit cans and obtain the reinforcement; once the reinforcement was received, the rat was immediately removed from the apparatus. During the 15-s interval between trials, rats were placed in a small Plexiglas box (50 cm × 30 cm × 30 cm). The average number of CR, RE, WE, and activity over the 3 days, integrated z-scores, and improvement scores were calculated.

##### 2.4.1.3 Simple visual task

Twenty-four hours after the end of the spatial task, rats were placed in the apparatus with the reinforced can having a different appearance (i.e., a Pepsi can) than the other identical six cans, and being randomly located on each trial. The simple visual task took place on 3 consecutive days and along 10 trials per day. As in the previous task, rats were allowed to spend up to 180 s per trial in order to visit cans and obtain water. During the 15-s interval between trials, rats were placed in the small Plexiglas box. The average number of CR, RE, WE, and activity over the 3 days, integrated z-scores, and improvement scores were calculated.

### 2.5 Barnes maze test

To assess spatial cognitive processing in an aversive context, the adolescent male rat offspring underwent the Barnes maze test (Gawel et al., 2019). Rodents find open, well-lit spaces aversive, thus they are motivated to search and localize an escape route (Sweatt, 2010). The apparatus consisted of a circular, grey platform made in Plexiglas (122 cm diameter × 90 cm height), with twenty holes with a diameter of 10 cm placed on the perimeter; one hole, namely, the target hole, led to an under-platform chamber (12 cm × 12 cm × 35 cm)—the escape box—while the other holes were covered underneath with a flat box and looked identical to the other. The location of the escape box varied according to the phase of the task. In the task, the animal was placed in the center of the platform and was initially unable to locate the escape box. Intense lighting—two points of light placed 1.5 m above the platform with a power of 500 W each—served as an additional stimulus during the task. On the walls of the laboratory room, large colorful geometric figures as visual cues were provided to facilitate the animal’s spatial orientation. The behavioral protocol consisted of the following phases—habituation; acquisition phase; probe task; reversal task (Gibula-Tarlowska et al., 2020)—as described below. In the probe task, the primary latency was recorded as the time required for the rat to make initial contact with the target hole to assess memory retention. The total distance traveled was recorded during the probe task as a measure of locomotor activity and exploratory behavior. The latency to escape—latency to find the escape box—was recorded during the reversal task. In order to outline the aversion-related cognitive performance, an integrated z-score was calculated (Guilloux et al., 2011; Castelli et al., 2022) based on the probe- and reversal tasks of the Barnes maze test, using normalization of primary latency and latency to escape. Individual aversion-related cognitive z-scores were then calculated by averaging z-score values. To calculate the recovery impact of CBD, the cognitive scores related to primary latency and latency to escape were calculated by subtracting the average of pTHC data (A) from both pTHC- and pTHC-CBD data (B) (delta = B-A) (Lima-Silva et al., 2012) multiplied by −1.

#### 2.5.1 Experimental design

##### 2.5.1.1 Habituation

Rats were placed in the middle of the maze and allowed to freely explore the apparatus for 180 s to habituate them to the platform and the escape box.

##### 2.5.1.2 Acquisition phase

The acquisition phase took place 24 hours after habituation. It consisted of 1 training session per day, 3 trials per session, for 4 consecutive days. The location of the escape box remained the same over all the acquisition trials. In order to assure that the initial orientation of the animal in the maze varies randomly across trials, rats were placed in the middle of the maze covered with an opaque bucket and let free, after a delay of a few seconds, to explore the platform. The trial was completed after 180 s or when the animal entered the escape box. Immediately after the animal entered the escape box, the hole was covered for 30 s. If the animal did not enter the escape box within 180 s, the experimenter gently guided it there.

##### 2.5.1.3 Probe task

Twenty-four hours after the acquisition phase, rats were placed in the maze where the target hole was closed. The reference memory of the location of the escape box was assessed for 90 s. Primary latency and total distance travelled were recorded; z- and improvement scores were calculated.

##### 2.5.1.4 Reversal task

Twenty-four hours following the probe task, the reversal task was performed. At that time, the position of the escape box was rotated 180° to the original, and three 180-s trials were run in 1 day (modified from Gibula-Tarlowska et al., 2020). The average score of latency to escape and z- and improvement scores were calculated.

### 2.6 Tissue collection and quantitative real-time polymerase chain reaction procedure

After the behavioral tests and 24 h after CBD administration, one male rat per litter, either from the Can test cohort or from the Barnes maze test cohort, was sacrificed and the brains were rapidly removed. Hippocampus was promptly dissected, flash-frozen in dry ice, and stored at −80°C until analysis. Homogenization in Trizol (Invitrogen) was performed to isolate RNA, followed by chloroform layer separation and isopropanol-induced precipitation. Ethanol washes (70%) were performed to remove residual salts from the isopropanol RNA precipitation step (Chomczynski and Sacchi, 2006). RNA was resuspended with water and then analyzed with NanoDrop (ND-1000 Spectrophotometer, Thermo Scientific, Wilmington, DE, United States). Afterward, RNA samples were reverse-transcribed to cDNA (SuperScript IV Reverse Transcriptase, Invitrogen), then, diluted and mixed with PowerUp SYBR Green Master Mix (Applied Biosystems) and primers. Samples were heated to 95°C for 10 m, followed by 40 cycles of 95°C for 15 s, 60°C for 1 m, 95°C for 15 s, 60°C for 30 s, and 95°C for 15 s. Analysis was performed using the delta-delta C (t) method. Primers employed are indicated in Table 1.

**TABLE 1:** Primers employed in qRT-PCR experiments.

Gene name	Primer sequence	Product
Gapdh	GTT​TGT​GAT​GGG​TGT​GAA​CC (Forward)	
	CTT​CTG​AGT​GGC​AGT​GAT​G (Reverse)	
NMDAR NR1 subunit - Grin1		Rn_Grin1_1_SG QuantiTect Primer Assay (QT00182287)
NMDAR NR2A subunit - Grin2A		Rn_Grin2a_1_SG QuantiTect Primer Assay (QT00379281)
PSD95 - Dlg4		Rn_Dlg4_1_SG QuantiTect Primer Assay (QT00183414)
mGluR5 - Grm5		Rn_Grm5_1_SG QuantiTect Primer Assay (QT01081549)
Homer 1 - HOM1	CTT​CAC​AGG​AAT​CAG​CAG​GAG (Forward)	
	GTC​CCA​TTG​ATA​CTT​TCT​GGT​G (Reverse)	
CB1R - Cnr1		Rn_Cnr1_1_SG QuantiTect Primer Assay (QT00191737)
Histidine triad nucleotide-binding protein 1 (HINT1)		Rn_Hint1_1_SG QuantiTect Primer Assay (QT01602713)

### 2.7 Statistical analysis

When data exhibited normality and equal variance, the difference between groups was determined by employing the Unpaired Student’s t-test and one-way analysis of variance (ANOVA) followed by the Bonferroni *post hoc* test, when appropriate. Nonparametric tests were performed if data did not show normal distribution or equal variance. Data are reported as mean ± SEM. Statistical analysis was performed using Prism v. 9 (GraphPad), and statistical significance was set at alpha = 0.05.

## 3 Results

### 3.1 CBD counterbalances the impaired cognitive score in the reinforcement-motivated task in pTHC-exposed adolescent rat offspring

**FIGURE 2:**
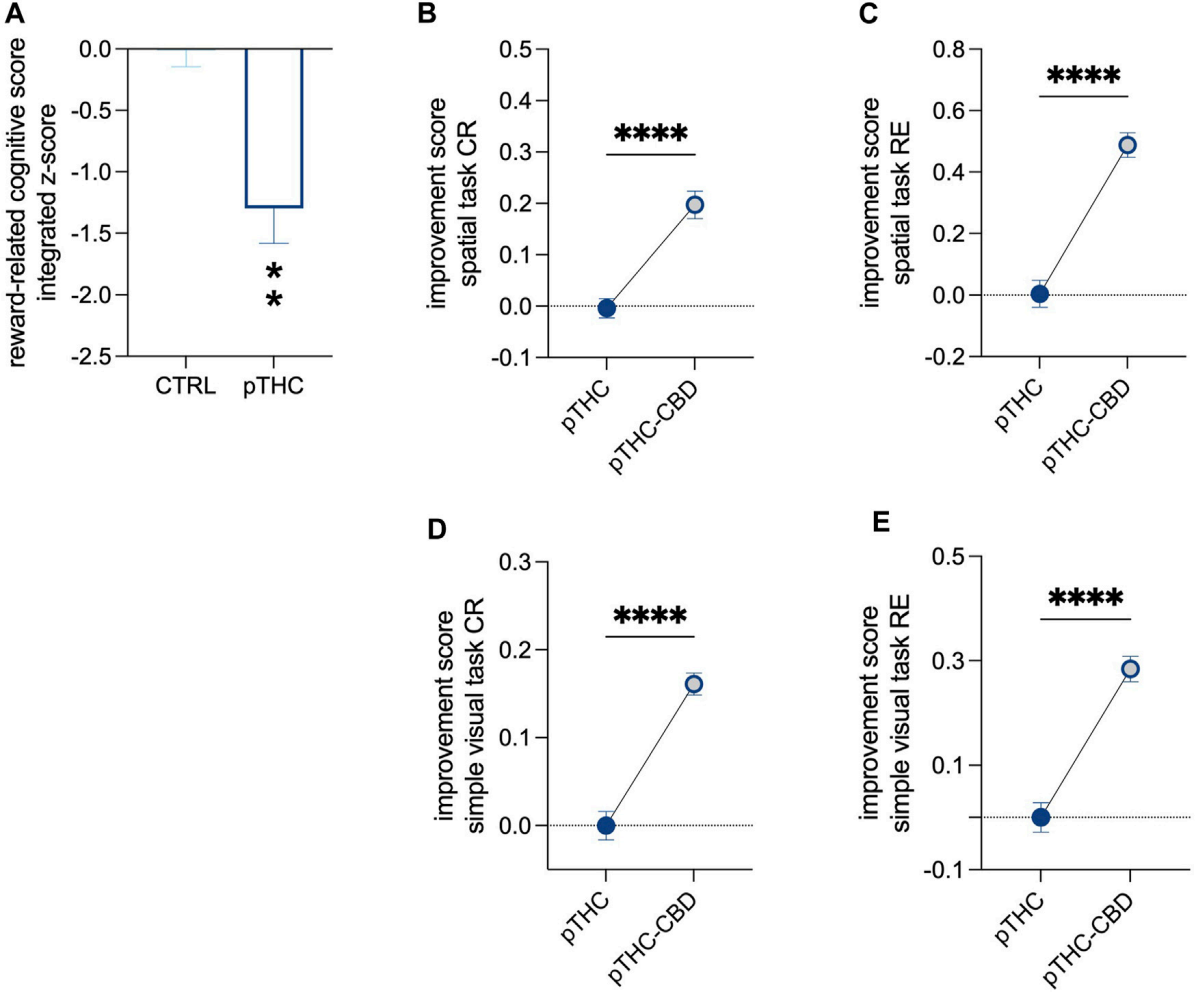
The recovery effect of CBD on the impaired reinforcement-related cognitive score of pTHC-exposed adolescent rat offspring. Rats prenatally exposed to THC displayed a lower reinforcement-related cognitive integrated z-score than CTRL rats **(A)**. CBD reversed pTHC-induced alteration in spatial and configural cognitive processing in the Can test of the adolescent offspring, by improving CR- and RE-related cognitive scores in pTHC-CBD rats tested in both spatial- **(B,C)** and simple visual (D,E) tasks. Each bar represents the mean of *n* = 10 rats; error bars indicate SEM. **p < 0.01, ****p < 0.0001. CTRL, male rat offspring prenatally exposed to Veh; pTHC, male rat offspring prenatally exposed to THC; pTHC-CBD, male rat offspring prenatally exposed to THC and exposed to CBD in adolescence; CR, correct responses; RE, reference memory errors.

When adolescent rat offspring were tested for spatial memory and the ability to discriminate objects independently of their position in the spatial memory acquisition- and the simple visual task of the reinforcement-motivated Can test (Figure 1), pTHC was shown to impair cognitive execution (Table 2). Indeed, the analysis of the reinforcement-related cognitive performance, with reference to both spatial- and configural memory in the reinforced context, indicated a significant effect of pTHC in decreasing the reinforcement-related cognitive integrated z-score (t = 4.085, df = 18, *p* = 0.0007; Figure 2A). CBD was able to reverse pTHC-induced alteration in spatial and configural cognitive processing of the adolescent offspring. Indeed, it induced a significant improvement in the CR- (t = 6.121, df = 18, *p* < 0.0001; Figure 2B) and RE- (t = 8.173, df = 18, *p* < 0.0001; Figure 2C)-related cognitive scores in pTHC-CBD rats tested in the spatial task. No significant effect was detected in the WE-related cognitive score (t = 0.5369, df = 18, *p* = 0.5979; data not shown) and in the activity score (KW = 5.865, *p* = 0.583; data not shown). Similarly, in the simple visual task, a significant improvement effect of CBD was detected in the CR- (t = 7.867, df = 18, *p* < 0.0001; Figure 2D) and RE- (t = 7.650, df = 17, *p* < 0.0001; Figure 2E) scores of prenatally THC-exposed offspring, while no impact was shown in WE-related cognitive score (U = 31, *p* = 0.1409; data not shown). No statistical test was performed for activity score in the simple visual task since all the values were the same.

**TABLE 2:** Effect of prenatal THC exposure (2 mg/kg) on spatial- and configural memory of the adolescent male rat offspring in the reinforcement-mediated Can test. Data are presented as mean ± SEM. CTRL, male rat offspring prenatally exposed to Veh; pTHC, male rat offspring prenatally exposed to THC; CR, correct responses; RE, reference memory errors; WE, working memory errors; n.s., non-significant.

		CTRL	pTHC	Statistics	
*Can test*					
spatial task	CR	0.56 ± 0.02	0.46 ± 0.02	Unpaired *t*-test	*p* < 0.01
	RE	0.82 ± 0.04	1 ± 0.04	Unpaired *t*-test	*p* < 0.01
	WE	0.04 ± 0.01	0.06 ± 0.02	Unpaired *t*-test	n.s.
	activity	9.97 ± 0.02	9.88 ± 0.05	Mann-Whitney test	n.s.
simple visual task	CR	0.83 ± 0.02	0.75 ± 0.01	Unpaired *t*-test	*p* < 0.001
	RE	0.23 ± 0.02	0.41 ± 0.03	Unpaired *t*-test	*p* < 0.0001
	WE	0.01 ± 0.00	0.01 ± 0.00	Unpaired *t*-test	n.s.
	activity	10 ± 0.00	10 ± 0.00	Mann-Whitney test	n.s.

### 3.2 CBD counteracts the impaired cognitive score in the aversion-related task in pTHC-exposed adolescent rat offspring

**FIGURE 3:**
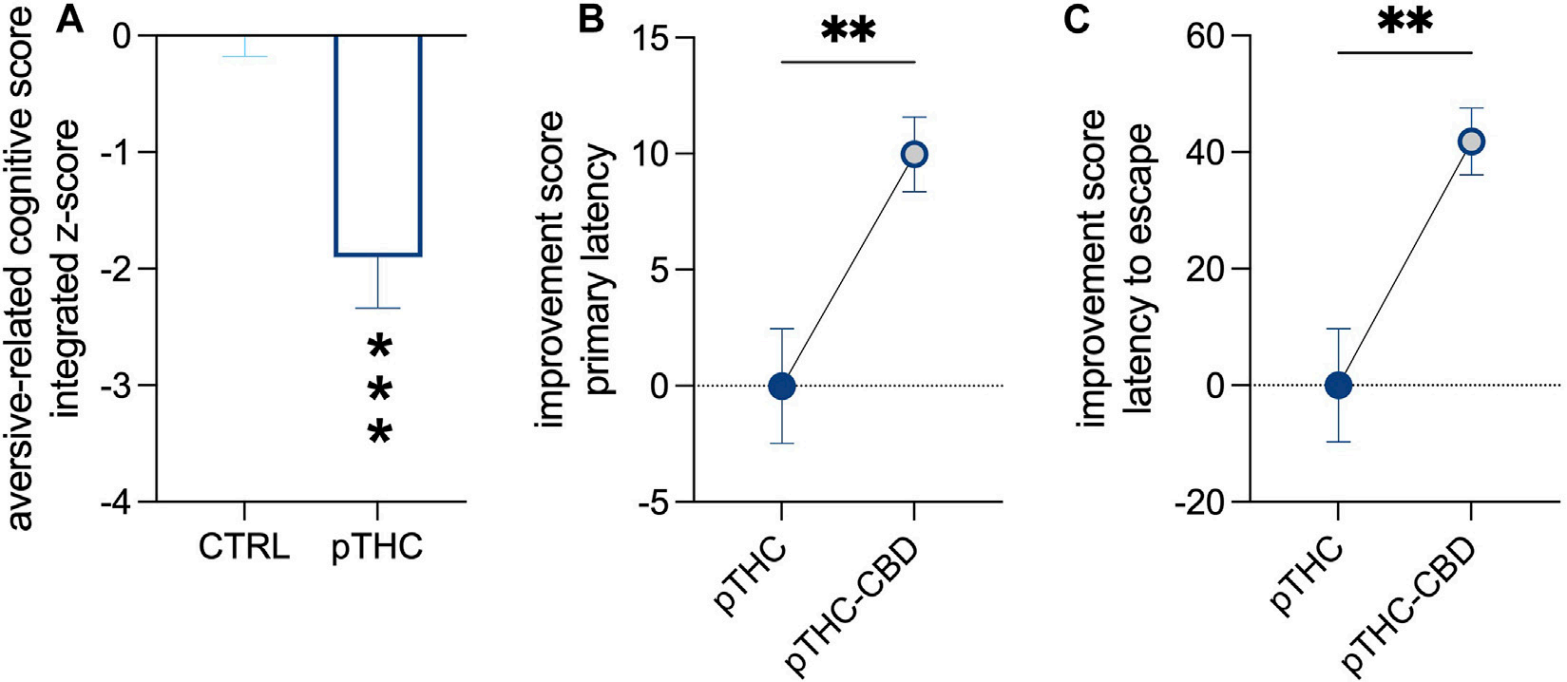
The ability of CBD to counteract the impaired aversion-related cognitive score of pTHC-exposed adolescent rat offspring. pTHC decreased the aversion-related cognitive integrated z-score **(A)**. CBD rescued pTHC-induced impairment in the retrieval of spatial memory and reversal learning, by improving primary latency- **(B)** and latency to escape-related cognitive scores **(C)** in pTHC-CBD rats. Each bar represents the mean of n = 12 rats; error bars indicate SEM. **p < 0.01, ***p < 0.001. CTRL, male rat offspring prenatally exposed to Veh; pTHC, male rat offspring prenatally exposed to THC; pTHC-CBD, male rat offspring prenatally exposed to THC and exposed to CBD in adolescence.

Rats were tested in adolescence for spatial memory and reversal learning in the aversion-driven Barnes maze test (Figure 1). Prenatal exposure to THC altered the execution of the probe- and reversal tasks (Table 3). Indeed, the analysis of the aversion-related cognitive performance, referring to both spatial memory retrieval and reversal learning in the aversive context, showed that pTHC significantly decreased the aversion-related cognitive integrated z-score (t = 4.057, df = 22, *p* = 0.0005; Figure 3A). CBD counteracted pTHC-induced impairment in the spatial cognitive processing of adolescent rat offspring. Indeed, CBD significantly improved the primary latency- (t = 3.225, df = 21, *p* = 0.0041; Figure 3B) and latency to escape- (t = 3.344, df = 22, *p* = 0.0029; Figure 3C) related cognitive scores in pTHC-CBD rats tested respectively in the probe- and reversal tasks of the Barnes maze test. No significant differences among the experimental groups were detected in the total distance travelled during the probe task (KW = 3.819, *p* = 0.1482; data not shown).

**TABLE 3:** Effect of prenatal THC exposure (2 mg/kg) on spatial memory and reversal learning of the adolescent male rat offspring in the aversion-motivated Barnes maze test. Data are presented as mean ± SEM. CTRL, male rat offspring prenatally exposed to Veh; pTHC, male rat offspring prenatally exposed to THC; n.s., non-significant.

		CTRL	pTHC	Statistics	
*Barnes maze test*					
probe task	primary latency	9.87 ± 1.73	16.87 ± 2.47	Unpaired *t*-test	*p* < 0.05
reversal task	latency to escape	39.29 ± 4.63	81.64 ± 9.69	Unpaired *t*-test	*p* < 0.05

### 3.3 CBD fine-tunes the abnormal gene expression of markers of excitatory plasticity and CB1R-related signalling in the hippocampus of pTHC-exposed adolescent rat offspring

**FIGURE 4:**
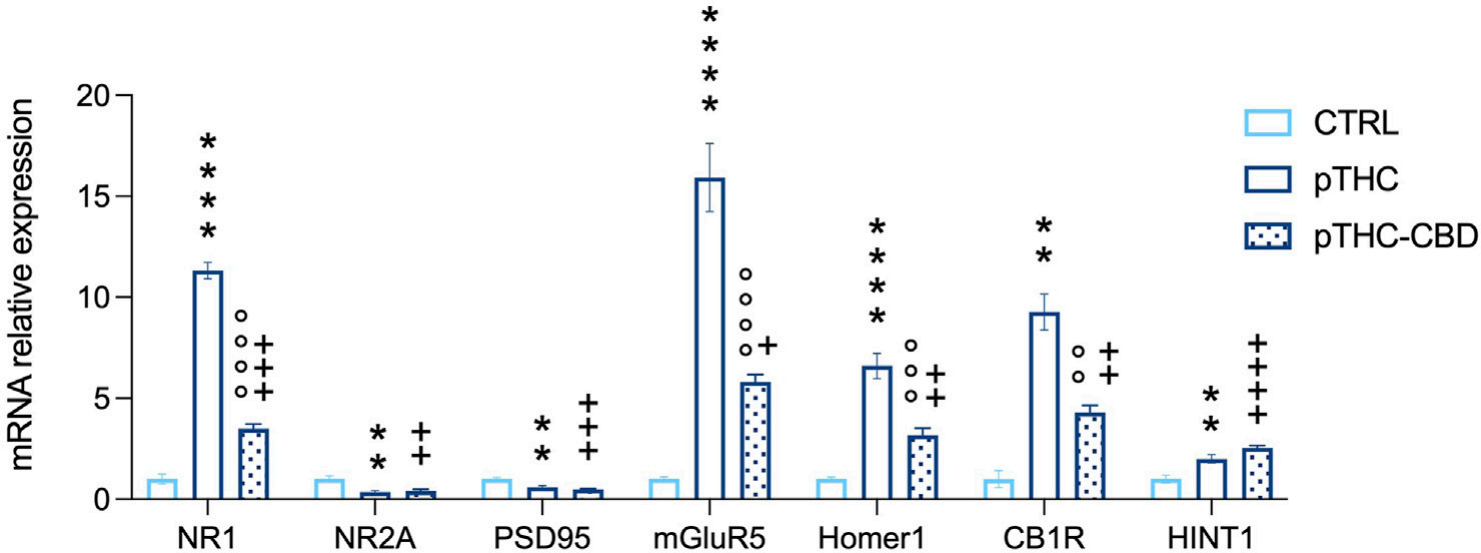
CBD (40 mg/kg) mitigates the abnormal mRNA relative expression levels of specific markers of excitatory plasticity and CB1R-related signaling in the hippocampus of pTHC-exposed (2 mg/kg, GD 5–20) adolescent rat offspring. Prenatal THC exposure increased the expression levels of the NMDAR NR1 subunit and decreased the levels of NR2A and PSD95, induced an increase in mGluR5, and its scaffolding partner Homer 1 isoform, and increased CB1R and HINT1 expression levels in the hippocampus of the adolescent male rat offspring. On the contrary, a decrease in NR1 subunit, mGluR5, Homer 1 and CB1R was detected in pTHC-CBD when compared to pTHC adolescent offspring. Each bar represents the mean of n = 6 rats; error bars indicate SEM.**pp < 0.01, ****pp < 0.0001, CTRL vs. pTHC; °°*p*p < 0.01, °°°*p*p < 0.001, °°°°*p*p < 0.0001, pTHC vs. pTHC-CBD; +*p*p < 0.05, ++*p*p < 0.01, +++*p*p < 0.001, ++++*p*p < 0.0001, pTHC-CBD vs. CTRL. CTRL, male rat offspring prenatally exposed to Veh; pTHC, male rat offspring prenatally exposed to THC; pTHC-CBD, male rat offspring prenatally exposed to THC and exposed to CBD in adolescence; NR1, ionotropic glutamate N-methyl-d-aspartate receptors (NMDARs) NR1 subunit; NR2A, NMDARs NR2A subunit; PSD95, scaffolding protein post-synaptic density-95; mGluR5, group I metabotropic glutamate receptor 5; CB1R, cannabinoid receptor type 1; HINT1, histidine triad nucleotide-binding protein 1.

We examined the mRNA relative expression levels of proteins playing a role in modulating the hippocampal excitatory synapse strength. The analysis of the expression levels of NMDAR subunit NR1, key for regulating the channel properties and the strength of the excitatory signaling, showed significant differences among the experimental groups (F = 309.8, *p* < 0.0001). Bonferroni *post hoc* analysis showed an increase in pTHC rats when compared with CTRL offspring (t = 23.86, DF = 15, *p* < 0.0001), while a decrease was detected in pTHC-CBD rats when compared to pTHC offspring (t = 18.08, DF = 15, *p* < 0.0001), although the gene expression levels remain higher than the control values (t = 5.775, DF = 15, *p* = 0.0001). In addition, when we considered the gene expression levels of the NR2A subunit, crucial for the NMDAR localization in the postsynaptic density and its functionality, significant group differences were detected (F = 9.626, *p* = 0.0020). In detail, pTHC induced a decrease in gene expression levels in comparison with CTRL rats (t = 3.970, DF = 15, *p* = 0.0037) which was not modified by CBD (pTHC-CBD vs. pTHC, t = 0.3674, DF = 15, *p* > 0.9999; pTHC-CBD vs. CTRL, t = 3.603, DF = 15, *p* = 0.0078). Similarly, the analysis of the expression levels of PSD95, the NR2A scaffolding protein critical for the PSD localisation of the NMDAR, showed significant differences among the groups (F = 17.95, *p* = 0.0001). Lower mRNA levels were detected in pTHC rats than in control counterparts (t = 4.561, DF = 15, *p* = 0.0011). CBD administration did not restore this alteration in pTHC offspring (pTHC-CBD vs. pTHC, t = 1.085, DF = 15, *p* = 0.8851; pTHC-CBD vs. CTRL, t = 5.646, DF = 15, *p* = 0.0001). Moreover, when the expression levels of the molecular effectors that prompt ECS-mediated long-term control of the synaptic strength were evaluated, the analysis showed significant group differences in mGluR5 mRNA relative expression levels (F = 58.18, *p* < 0.0001). Bonferroni *post hoc* test showed an increase in mRNA relative expression in pTHC rats when compared with CTRL offspring (t = 10.57, DF = 15, *p* < 0.0001), while a decrease was detected in pTHC-CBD rats when compared to pTHC counterpart (t = 7.163, DF = 15, *p* < 0.0001), although the gene expression levels remain higher than the control values (t = 3.404, DF = 15, p 0.0118). Likewise, when we analysed the expression levels of the scaffolding protein Homer 1, whose binding to mGluR5 is critical for evoking ECS-mediated LTD, we detected significant group differences (F = 45.07, *p* < 0.0001). In detail, the analysis highlighted an increase in gene expression levels in pTHC rats compared with CTRL rats (t = 9.415, DF = 15, *p* < 0.0001) that were mitigated by CBD (t = 5.765, DF = 15, *p* = 0.0001), although they remained higher than control levels (t = 3.650, DF = 15, *p* = 0.0071). Moreover, we explored the expression levels of CB1R in the hippocampus: the analysis showed significant differences among the groups (F = 47.62, *p* < 0.0001). Indeed, an increase in CB1R gene expression was detected in pTHC rats when compared with the CTRL group (t = 9.693, DF = 15, *p* < 0.0001), which was decreased by CBD (t = 5.827, DF = 15, *p* < 0.0001), although the gene levels remain higher than the control values (t = 3.866, DF = 15, *p* = 0.0046). On the other hand, when we evaluated the mRNA expression of HINT1, a functional effector of the CB1R, the analysis showed significant differences among the groups (F = 18.95, *p* < 0.0001), with levels being higher in pTHC offspring than in control rats (t = 4.146, DF = 15, *p* = 0.0026); CBD did not modify pTHC-induced alterations in HINT1 gene expression levels (pTHC-CBD vs. pTHC, t = 1.868, DF = 15, *p* = 0.2442; pTHC-CBD vs. CTRL, t = 6.014, DF = 15, *p* < 0.0001) (Figure 4).

## 4 Discussion

The present research confirms and extends growing evidence on the detrimental effect induced by THC prenatal exposure on the behavioral and molecular correlates of spatial and configural memory of adolescent male rat offspring. Furthermore, for the first time, our data reveals a significant activity of CBD in improving the memory scores and fine-tuning the molecular markers of hippocampal synaptic plasticity and eCBs signaling of pTHC-exposed adolescent rats.

Animals, including rats, are predisposed to process and use spatial information and configural association to organize and guide behavior. Prenatal exposure to THC impaired spatial and configural memory both in the reinforcement-motivated context of the Can test and in the aversive setting of the Barnes maze test. Indeed, we measured a deflection in the integrated score in which indices of spatial-, configural memory and reversal learning were taken into account. The effect of pTHC exposure, in our experimental conditions, seems to involve specific microcircuits for spatial- and configural memory in the adolescent male offspring since the occurrence of decreased incentive drive or emotional dysregulation did not characterize the pTHC-exposed offspring’s phenotype (Castelli et al., 2023). These results are consistent with human data from the adolescent offspring of mothers exposed to THC who exhibited deficits in the consolidation and retrieval of both verbal and visual information, reversal learning, and visual discrimination (D’Souza et al., 2004; Goldschmidt et al., 2000; Pope and Yurgelun-Todd, 1996). It is largely reported that THC-induced impairment in memory formation in emotionally salient contexts occurs by the activation of CB1Rs (Wise et al., 2009; Brancato et al., 2016), confirming a selective role of hippocampal CB1R-related signaling in the regulation of cognitive processing. Overall, our data indicates that in-utero exposure to THC elicits a supraphysiological impact on the ECS that may result in a complex, specific interaction with the developing brain, and can change the normative trajectory of cellular processing and neurocircuitry that underlines the formation of spatial- and configural memory (Bara et al., 2021). This can explain not only the impaired cognitive performance of the adolescent rats but also the molecular abnormalities in the relevant players of the excitatory synapse and eCBs signalosome observed in the hippocampus of the adolescent male rat offspring. In detail, prenatal THC exposure set out an excessive expression of NMDAR NR1 subunit, and dampened levels of NR2A subunit- and PSD95 mRNA expression, suggestive of a profound disturbance in the excitatory synapse, which can be functionally related to the observed behavioral outcomes. Evidence has confirmed that maternal exposure to cannabinoids affects the proliferation, migration, and differentiation of glutamatergic and GABAergic neurons (Saez et al., 2014). Intriguingly, pTHC exposure is able to specifically decrease the population of inhibitory (cholecystokinin-positive) interneurons in the hippocampus, where CB1Rs are mainly expressed: this could design a dysfunctional microcircuit activity that by affecting the significant players of synaptic plasticity would produce an imbalance in the excitatory/inhibitory tone (Bateup et al., 2013; Bonansco and Fuenzalida, 2016). On the other hand, the overexpression of mGluR5/Homer 1 signaling and CB1R/HINT1 cooperation here reported, might be functionally interpreted as an attempt to mitigate the dysfunctional glutamatergic output promoted by pTHC exposure (Castelli et al., 2023). These two systems are in fact considered proxies of the inhibition of synaptic strength and NMDAR hyperactivity promoting, respectively an increase in eCBs production, and the removal of NR1 subunit’s excess. Indeed, pTHC exposure, by impinging on the organization of fetal cortical circuitry, is able to curtail eCBs bioavailability and CB1R mRNA expression during fetal development (Tortoriello et al., 2014; de Salas-Quiroga et al., 2015). In this regard, it seems likely that the observed upregulation of CB1R mRNA in adolescence may compensate for otherwise impaired endocannabinoid signaling (Frieling et al., 2009).

As much as the gestational epoch is particularly sensitive to developmental threats (Levendosky et al., 2021), adolescence is a time of high neural plasticity and a unique window of opportunity for “brain reprogramming” (Levendosky et al., 2021; Tymofiyeva and Gaschler, 2021). Our hypothesis, in fact, is that the aberrant cognitive trajectories produced in the prenatal period by THC exposure could be rescued by the wide-spectrum activity of CBD. Indeed, CBD administration has been reported to improve cognitive deficits in several domains, which may vary depending on the pathological condition and/or the dosage (Esposito et al., 2011; Campos et al., 2015; Peres et al., 2016; García-Baos et al., 2021; Meyer et al., 2021; Niloy et al., 2023). In our experimental setting, CBD administration, before the test sessions, normalized the impaired cognitive scores of the adolescent male rat offspring exposed prenatally to THC, independently from the emotional/motivational salience of the task. In the reward-motivated Can test locomotor activity and the incentive drive support the animals to achieve the reinforcement. CBD, however, did not change the exploratory drive to search for the reinforced can, as shown by the unaltered activity score, suggesting that CBD may specifically modulate memory processing. On the other hand, the Barnes maze harnesses the natural preference of rodents for dark and quiet environments, thus, the aversive cues—i.e., bright light and open spaces—may provide an anxiogenic setting that increases the motivation to search the escape box (Gawel et al., 2019). Therefore, the performance of rodents in the Barnes maze may be influenced by non-cognitive factors, such as the stressing environment (Gawel et al., 2019). However, the lack of difference in locomotor activity and exploratory behavior between the experimental groups in the probe task of the Barnes maze test suggests that the CBD-enhancing effect on the cognitive score does not rely on its interference with rat behavioral reactivity. Accordingly, although the popularity of CBD is rapidly escalating for its anxiety-, and stress-reduction potential (Blessing et al., 2015), previous evidence indicates that anxiety-related tasks, such as risk assessment, are not significantly sensitive to CBD (Twardowschy et al., 2013). Furthermore, CBD administration is shown not to impact anxiety-like behavior in the elevated plus maze and spatial memory in the Barnes maze in control mice (Kaplan et al., 2021). Accordingly, the treatment with CBD 40 mg/kg did not alter the total locomotor activity of rats tested in actimeter infrared chambers (Umpierrez et al., 2022) and did not influence distance traveled, time spent in open arms, and open arms entries in an elevated zero maze (Skully, 2021). However, there is conflicting evidence from human and animal research that shows CBD may exert both anxiolytic and anxiogenic effects, depending on the application of different designs, types of tasks, and, thus, the functions they relate to (Fusar-Poli et al., 2009; Bhattacharyya et al., 2010; Marco et al., 2011). Notably, in our experimental conditions, CBD did not significantly modify the cognitive performance and the exploratory drive of the control offspring in the reinforcement- and aversion-motivated tasks. This evidence is supported by studies showing no independent beneficial effects of CBD on cognition, including verbal learning and memory, social recognition, executive function, spatial memory, or conditioned learning when administered to healthy subjects (humans or rodents); on the other hand, CBD is able to rescue the cognitive performance of adolescents after THC administration (Weston-Green, 2019). The ability of CBD to rescue cognitive decline has been shown in neurodegenerative- and psychiatric disorders and brain damage (Esposito et al., 2011; Campos et al., 2015; García-Baos et al., 2021). Intriguingly, the neuroprotective effect of CBD has been also demonstrated in a rat model of cognitive impairment by neonatal oxidative damage, with a significant improvement of recognition indexes in a long-term retention test, suggesting that CBD might rescue memory impairments by reversion/prevention of oxidative stress in brain regions relevant to memory formation (Fagherazzi et al., 2012).

In line with the rescue effect on the deteriorated cognitive score, CBD discretely scaled down the over-expression of the markers of synaptic plasticity in the adolescent rat offspring following pTHC exposure. In detail, CBD significantly downregulated the abnormal increase in NMDA NR1 subunit expression but did not produce alterations in PSD levels. Notably, CBD effect on the expression of the NMDAR subunits was previously described by Mao and others (Mao et al., 2015) who observed a marked decrease in NR1 mRNA level in the rat hippocampus in a model of chronic epilepsy supporting CBD therapeutic activity in conditions of neuronal hyperexcitability. Indeed, CBD has been reported to protect neurons against glutamate excitatory signaling, representing a potentially useful medication for a diverse range of neurological disorders (Pretzsch et al., 2019). Moreover, in our study, CBD mitigated the overexpression of mGluR5/Homer 1 levels, likely reducing the 2-arachidonoylglycerol (2-AG) (rather than anandamide)-mediated retrograde synaptic plasticity known as metabotropic suppression of excitation. This is in accordance with CBD being reported to inhibit mGluR group I agonist-mediated suppression of excitation in a model of autaptic hippocampal neurons (Straiker et al., 2018). If, as it is reported, the activation of the mGluR5/Homer 1 system activates 2-AG-driven depression of the synaptic strength in order to dampen NMDAR-related excitatory signaling, therefore the double balancing effect exerted by CBD on the NR1 and mGluR5/Homer 1 components may contribute to the recovery of synaptic efficiency. Notably, we also report that CBD administration was associated with a decrease in hippocampal CB1R mRNA expression in the adolescent pTHC offspring. CBD activity on the ECS covers wide-spectrum effects, and among these, an increase in endogenous anandamide (AEA) levels, mainly through the inhibition of the enzyme fatty acid amide hydrolase (FAAH), plays a crucial role (De Felice and Laviolette, 2021). In support of this finding, the administration of the FAAH inhibitor URB597 in adult female rats was able to rescue cognitive and depressive-like symptoms induced by adolescent THC exposure (Bhattacharyya et al., 2010; Realini et al., 2011). On this basis, CBD-induced higher availability of AEA would, therefore, cause a reduction in the expression of CB1Rs as a homeostatic response (Guagnini et al., 2006; Frieling et al., 2009; Hirvonen et al., 2012), in agreement with some previous studies demonstrating a CBD/AEA-recovery effect associated with a reduction in CB1R mRNA expression in different brain regions (Portugalov et al., 2022). In this regard, the recent finding that CBD has a profile consistent with a negative modulation of CB1R signaling both at a cellular and nuclear level (Laprairie et al., 2015) offers a significant and welcome insight. Interestingly both AEA and CBD act as direct agonists of TRPV1 (and TRPV2) channels (Bisogno et al., 2001; Muller et al., 2021); TRPV1, in particular, is ideally located in the hippocampal CA1 region to modulate excitatory glutamate signaling. Intriguingly upon the following exposure, CBD rapidly desensitizes the TRPV1 channel (Anderson et al., 2022) and, thus, may reduce neuronal hyperexcitation (Chávez et al., 2010; Etemad et al., 2022). Overall, given the evidence of pTHC-induced disturbances in the normative development of hippocampus-related cognitive functions, here we propose that CBD treatment during adolescence, by its multitarget activity, can mitigate the abnormal excitatory/inhibitory tone and downregulate the effectors of eCBs-driven LTD, which underlying impaired cognitive processing in pTHC-exposed rats. Actually, multiple other receptors, channels, and systems can be involved as putative targets for CBD buffering activity in our experimental conditions, such as, but not only, adenosine-, GABA-A, and serotonin receptors (Sylantyev et al., 2013; Aso et al., 2019; De Gregorio et al., 2019). Indeed, we do not aim at reviewing CBD pharmacological properties (for a complete review see Castillo-Arellano et al., 2023), although the investigation of the causal interpretation of the current results is in progress.

## 5 Conclusion

This research adds significant behavioral and molecular evidence to the increasing reports of pTHC- induced detrimental effects on cognitive functions, and stresses the importance of a collective endeavor to curb gestational cannabis use (Groff et al., 2023). Accordingly, the evaluation of a safe and effective strategy to rescue pTHC-induced impairment is a research priority. The therapeutic potential of CBD has only began to be revealed. Growing evidence suggests that CBD exhibits promising therapeutic activity in epileptic seizures, autism, sleep disturbances, chronic pain, and in the context of addiction (Bilge and Ekici, 2021; Shannon et al., 2019; Boyaji et al., 2020; Maniaci et al., 2015; Prud’homme et al., 2015). Indeed, recent data shows that CBD can mitigate the detrimental effects of THC consumption in adolescence. Specifically, preclinical studies suggest that THC-induced immediate and long-term impairments in working memory in adolescence and the long-term changes in cortical molecular components were recovered by the co-administration of CBD, whereas CBD as a single agent did not generate behavioral outcomes (Murphy et al., 2017). Here, we show for the first time that CBD administration in adolescence represents a strategy to mitigate pTHC-induced harm in the progeny since it improves the memory scores and fine-tunes relevant effectors of hippocampal plasticity. The molecular mechanism by which CBD appears to counterbalance pTHC effect on the developing brain in involves the modulation of the hippocampal synaptic strength, during the sensitive time window of adolescence. However, considering that CBD modulates the activity of proteins involved in neuronal pathfinding and maturation, such as CB1Rs, we still do not know what the impact of high-dose CBD may be when administered during early neurodevelopment. Further preclinical evaluation covering different epochs of neurodevelopment is required to consolidate the safety and therapeutic properties of CBD.

## Data Availability

The raw data supporting the conclusion of this article will be made available by the authors, without undue reservation.
